# Oral Mucosa in Cancer Patients—Putting the Pieces Together: A Narrative Review and New Perspectives

**DOI:** 10.3390/cancers15133295

**Published:** 2023-06-22

**Authors:** Jose Manuel Reuss, Laura Alonso-Gamo, Mariola Garcia-Aranda, Debora Reuss, Manuel Albi, Beatriz Albi, Debora Vilaboa, Beatriz Vilaboa

**Affiliations:** 1 Department of Postgraduate Prosthodontics, Universidad Complutense de Madrid, 28040 Madrid, Spain; 2 Department of Pediatrics, Hospital Infanta Cristina, 28981 Madrid, Spain; 3 Centro Integral Oncológico Clara Campal, Department of Oncologic Radiotherapy, Hospital Universitario Sanchinarro, 28050 Madrid, Spain; 4 Lecturer Dental School, Universidad San Pablo CEU, 28003 Madrid, Spain; 5 Department of Gynecology and Obstetrics, Quironsalud Group Public Hospitals, 28223 Madrid, Spain; 6 Department of Gynecology and Obstetrics, Hospital Universitario Fundación Jiménez Díaz, 28040 Madrid, Spain; 7 Aesthetic Dentistry Department, Universidad San Pablo CEU, 28003 Madrid, Spain; 8 Clínica Vilaboa, 28001 Madrid, Spain

**Keywords:** mucositis, oral mucosa, saliactive, antioxidant, microbiome boost, cancer toxicity, vagina, breast cancer survivors, head and neck cancer, cancer support

## Abstract

**Simple Summary:**

The oral mucosa, as part of human mucosa, is an essential part of the oral cavity. It is the first natural barrier against the intrusion of newcomers and a habitat for the oral microbiome. As such, it is receiving ever-increasing interest in clinical and translational research. However, it is in the field of cancer treatment and its toxicity wherein oral mucosa has a prominent role, as oral mucositis is one of the most debilitating cancer treatment complications and a notorious source of worry for oncologists and poor quality of life for cancer patients. This narrative review focuses on the entangled implications of human oral mucosa in cancer and, specifically, in the development of cancer-related oral mucositis support strategies.

**Abstract:**

The oral mucosa is a key player in cancer patients and during cancer treatment. The increasing prevalence of cancer and cancer-therapy-associated side effects are behind the major role that oral mucosa plays in oncological patients. Oral mucositis is a debilitating severe complication caused by the early toxicity of chemo and/or radiotherapy that can restrict treatment outcome possibilities, even challenging a patient’s survival. It has been referred to as the most feared cancer treatment complication. Predictive variables as to who will be affected, and to what extent, are still unclear. Additionally, oral mucositis is one of the sources of the increasing economic burden of cancer, not only for patients and their families but also for institutions and governments. All efforts should be implemented in the search for new approaches to minimize the apparently ineluctable outburst of oral mucositis during cancer treatment. New perspectives derived from different approaches to explaining the interrelation between oral mucositis and the oral microbiome or the similarities with genitourinary mucosa may help elucidate the biomolecular pathways and mechanisms behind oral mucosa cancer-therapy-related toxicity, and what is more important is its management in order to minimize treatment side effects and provide enhanced cancer support.

## 1. Introduction

The mucosa, also called the mucous membrane, is one of the most extensive organs in the human body, which is found mainly in the oral cavity, gastrointestinal tract, urogenital tract, respiratory tract, and, to a lesser extent, in organs such as the eyes. It behaves as a defensive barrier, resists physical stimuli and friction, and, therefore, has a high rate of cell turnover. It also has absorptive functions and carries a rich microbiota, which gives it unique properties in health and well-being.

The oral mucosa is the specialized part of the mucosa that covers the entire oral cavity up to the oropharynx. Histologically, it consists of a stratified squamous epithelium and an underlying connective tissue or lamina propria, which is keratinized at sites of friction. In the area where it meets the teeth, it differentiates into a masticatory mucosa that is tightly adherent to the underlying bone, known as attached gingiva. 

Furthermore, the function that has received the most attention recently is its immunological role, in addition to serving as a preferential habitat for the oral microbiome, as mentioned previously [1].

## 2. Oral Mucosa and the Barrier Function

One of the main roles of the mucosa is its barrier function. An ample diversity of challenges can disrupt the epithelial barrier of the mucosa. Among them are end metabolites and allergens derived from food, alcohol, therapeutic agents, inadequate oral hygiene, bacteria, virus, and fungi. Dysregulation or unbalance in the epithelial barrier has been described as the origin of a leaky epithelium that generates dysbiosis through a double mechanism, first, reducing commensal microbes and second, enhancing opportunistic pathogens. Said damage to the mucosal barrier can be exerted at different levels in the epithelium, such as the zonula occludens 1, 2, and 3, claudins, E-cadherins, desmosomes, and junctional adhesion molecules [2]. The breakage of the barrier is behind the crossing of species that penetrate the mucosa barrier and exert their different signals, inflammatory, carcinogenic, or infectious, as well as immune system downregulation pathways. Species penetration in cancer patients has a recently described double impact: the initiation and progression of the cancer and the modulation of the treatment outcome [3].

The role of the oral mucosa barrier in both the pathogenesis of and resilience to COVID-19 infection is still under study. The preferential invasion of the virus through the oral mucosa is explained by the fact that it is a receptacle for a high number of ACE2 receptors throughout the oral mucosal epithelium that are used as couplings by viral particles, allowing the release of key parts of the virus into the cell cytoplasm for replication [4]. The elevated presence of ACE2 receptors in oral mucosal cells means that the oral mucosa should be considered an area of high risk for SARS-CoV-2 adhesion and penetration [5].

Lesions and manifestations of SARS-CoV-2 in the oral mucosa include dryness of the mucosa, mucosal irritation, and inflammation and pain caused by the release of tumor necrosis factors and cytokines associated with the acute inflammatory episode characteristic of this viral infection [6].

Taste and smell reside primarily in the oral mucosa, in the taste receptors on the dorsum of the tongue (taste buds), in the tonsils and oropharynx, and in the olfactory receptors located in the nasopharynx. Both can be altered or markedly diminished in COVID-19. The loss of taste and smell (dysgeusia and anosmia) is often sudden and abrupt. Mucosal congestion or edema and mucosal dryness may explain the loss of smell [7], while the coexistence of most of the taste buds on the dorsum of the tongue, particularly in the area with the highest concentration of ACE2 receptors, and their involvement in the virus entry and local destruction mechanisms and, therefore, being heavily exposed to the cell damage caused by the virus, may be the origin of the loss of taste [8,9]. Taste and smell are also claimed by patients to change during cancer treatment, as the oral mucosa is especially susceptible to chemo and radiotherapy [10].

## 3. Oral Mucosa and the Toxicity of Cancer Treatment

The oral mucosa is a main actor in cancer. The toxicity of cancer treatment rapidly and preferentially affects the oral mucosa, causing oral mucositis. 

Oncological-treatment-induced oro-gastro-intestinal mucositis, otherwise recognized as oral mucositis, or mucositis, is the most visible clinical complication of cancer therapy and one of the most feared by oncologists [11]. It has been described as the hidden side of cancer treatment, with varying incidence rates depending on the type of cancer and the mode of therapy (Table 1) [12]. Oral mucositis is highly prevalent not only in the treatment of head and neck cancer but also in the treatment of any type of cancer [13,14,15]. Mucositis can affect the oral mucosa throughout the whole of the oral cavity, the throat, the larynx, and also the digestive tract including the rectum and anus, the respiratory tract, as well as the vagina [16]. 

Regarding the type of agent, it has been described that, specifically, H&N patients treated with cisplatin plus radiotherapy will develop oral mucositis [17]. Taxanes and their derivatives usually generate a milder grade of mucositis, but docetaxel is associated with a higher risk of developing mucositis in comparison with paclitaxel [18]. Women treated with 5-fluoracil (5-FU) for colorectal carcinoma experience higher severity grades, more types of toxicity, and a higher incidence of severe toxic effects, showing gender differences in 5-FU-related toxicity independent of the treatment regimen, patient characteristics, and cancer trial method [19].

New treatment regimens, such as the mammalian target of rapamycin (Mtor-inhibitor), exert high mucosal toxicities with different characteristics from typical oral mucositis. It can be recognized via a necrotic area surrounded by erythema, which produces very significant pain that frequently obliges the remodulation of the therapy [20,21,22]. Epidermal growth factor (EGFR) inhibitors produce mucosal damage in 15% of patients [23]. The mucosa lesions look like aphthous ulcers. Monoclonal antibodies addressing the vascular endothelial growth factor receptor (VEGFR) very seldom produce any type of mucositis/stomatitis [24].

Oral mucositis lesions vary from erythema to ulceration in the mucosa developing with different grades of pain, swallowing, eating, and speaking impairment, compromised nutrition, weight loss, and dehydration, with treatment delays and even interruptions and hospitalizations. Mucosa ulcerations and inflammation can be accompanied by bleeding and pain and may impair specific functions depending on the sites affected. Mucosal barrier disruption leads to pathogen penetration and potentially systemic bacteriemia and sepsis that may be fatal. Oral mucositis has a significant impact on the patient’s quality of life and may negatively impact the survival of the cancer patient [25]. The well-described pathophysiologic stages of the mucosal barrier disruption that takes place in the oral mucosa during the outburst of oral mucositis episodes are well understood, following the five-stage model that has already been described in 2004 [26,27]. It starts with the first stage of initiation, the first step, the second being the up-regulation and generation of messenger signals, followed by the third stage of signaling and amplification mediated by pro-inflammatory cytokines, producing the ulceration stage in the fourth phase in which colonization by Gram-positive, Gram-negative, and anaerobes takes place, progressing into a final healing phase referred to as the fifth step, with the renewal of the different affected layers and the recovery and reestablishment of the local microflora, in the words of the authors. Interestingly, they explain that despite the five-step model referring to a linear cascade of events, the fast-occurring damage happens simultaneously throughout the whole tissue. Even though the cellular damage was explained as being linked to the generation of oxidative stress and reactive oxygen species (ROS), the implication of inflammatory mediators in eliciting mucosal damage cannot be out-ruled. It is worth mentioning that the authors refer to the implication of the local microflora that reestablishes itself during the healing process. 

The interest in the responsibility of bacteria in the pathogenesis of oral mucositis has resulted in numerous papers [28,29,30,31] that have elucidated the role of the oral microbiome in oral mucositis. However, the management of mucositis with antibiotics is unfruitful, which does not mean that bacteria change the course of the condition [32].

Thanks to the combination of metagenomics science with computational models, the physiopathologic understanding of oral mucositis has seen an evolution from the classical five-step model described by Sonis. The actual school of thought hypothesizes that there is a gene pathway implication of oral microbiome dysbiosis in the genesis of mucosal ulceration in oral mucositis and delayed and/or impaired wound healing in oral mucositis [33,34]. In other words, the mucosal barrier breakage is itself part of mucosal dysbiosis.

Cancer treatment strategies are designed to target rapidly dividing cancerous cells. Subsequently, tissues characterized by a high cell turnover such as the oral mucosa and gastrointestinal and vaginal mucosa are also subjected to chemotherapeutic agents through the cytotoxic effects of cancer therapy. The oro-gastro-intestinal mucosa is moist and elastic and exposed to friction (masticatory function and food transit) and therefore exhibits intense and constant cell turnover with high mitotic activity in cells and cell shedding every 14–21 days. This active cell turnover and metabolism is higher in the mobile mucosa and lower in the masticatory mucosa [35]. This particularity makes the oral mucosa very sensitive to antineoplastic treatments that are meant to target cancer cells with a high proliferative capacity.

The oral microbiome that is attached to mucosa cells, as well as the microbiome-related host defense and the crosstalk between the oral mucosa and the oral microbiome, is affected during oral mucositis. The novel discovery of the roles of major virulence factors from members of the oral microbiome in the development of oral mucositis, the duration of ulcerations, and the delay of tissue healing need to be further explored in order to better understand the relationship between oral mucositis and the oral microbiome, including the microbiota [3,36]. Whether it is the chicken or the egg, dysbiosis being the consequence of oral mucositis or the latter being the consequence of the former, is still unclear. What happens first in oral mucositis, chemo-radiotherapy-mediated cell death or oral-dysbiosis-related mucosal barrier disruption, is currently attracting the interest of researchers and clinicians [37,38].

## 4. Oral Mucositis and Oral Microbiome Dysbiosis

Dysbiosis has been reported to exist whenever disruption of the epithelial barrier occurs [39,40].

Patients immersed in cancer treatment suffer from this newly coined cancer dysbiosis [41]. Cancer-therapy-induced dysbiosis has been found in different types of cancers [42]. Furthermore, it has been outlined that it could well be that an altered microbiota can enrich the list of predisposing risk factors for breast cancer [43].

Cancer treatment changes the oral microbiome. At present, the hypothesis that the microbiota exerts a fundamental role not only in the cancer onset but also in the cancer treatment response is explained by the fact that certain species are behind in delayed and reduced wound healing [44]. For instance, *P. gingivalis* can inhibit wound healing and is able to escape the host immunity [45]. 

Oral microbiome sampling displays specific signatures associated with different types of cancer, for example, a reduction in the Streptococcus and *Rothia* species in oral cancer [46], reduced diversity and lower counts of streptococcus in lung cancer [47], reduced *neisseria* and reduced *streptococcus* in pancreatic cancer [48], and an enriched oral microbiota as an early marker of pancreatic cancer [49].

It has been demonstrated that subjects with *P. gingivalis* and periodontitis are at higher risk of pancreatic cancer [50]. *P gingivalis* has been appointed as a protagonist in oral dysbiosis in manifold papers. Its role in cancer and in oral mucositis is currently thought to be more of a principal role than one of a passive kibitzer.

In oral mucositis, the chemotherapy-disrupted oral microbiota is shifted to Gram-negative bacteria such as the *Fusobacterium nucleatum*, *Clostridia*, and *Treponema* species that are typically found in periodontitis and inflammatory conditions, while commensal subspecies such as Streptococcus are diminished. Dysbiosis with high counts of *F. nucleatum* has been associated with severe mucositis via the aggravation of epithelial injury and enhanced inflammation and apoptosis [37,51].

Gram-negative species and the above-mentioned mucositis-associated microbiome perpetuate themselves in the pro-inflammatory breeding ground, so all means of reversing or modulating oral dysbiosis could prove beneficial to cancer patients.

In view of the current evidence showing the implications of certain microbiota hubs, such as the gut microbiota, in the response to radiotherapy-induced mucositis, it seems at least sensible, if not wise, to seek the reintegration of a balanced oral microbiome and an oral microbiome boost in order to try to modulate the host local response to cancer therapy toxicity, namely, a lesser degree of oral mucositis [52].

## 5. The Example of Head and Neck Cancer

In 2020, global cancer statistics estimated 932,000 new cases of head and neck cancer (H&N) and 467,000 deaths globally [53]. The main risk factors have historically been alcohol and tobacco. However, in recent decades, the incidence of tobacco-related cancer has decreased, while in recent years, human papillomavirus (HPV) has become a major causative factor provoking more H&N cancers than cervical cancers [54]. In p16-positive oropharyngeal subjects, the disease can occur at young ages, creating a population of cancer survivors with potentially significant treatment-related morbidities for decades.

The current reality for H&N patients is one of younger subjects with higher cure rates, increasing numbers of survivors, increasing numbers of years as survivors, and multimodality management including chemotherapy and/or radiotherapy and/or surgery.

The most prominent cancer-therapy-related toxic effects according to the time of onset are the following:

1. Acute (during treatment): oral mucositis.
2. Chronic (after the end of treatment): xerostomia, dysphagia, pain, and persistent/chronic oral mucositis.

Patients may experience pain, social and physical limitations, psychological effects related to these, as well as self-appearance issues that altogether may lead to anxiety, depression, and lack of motivation to enjoy life. Unfortunately, these complications may be present “per se” in an acute, chronic, and long-lasting manner [55]. The quality of life of survivors has been described to be poor with more than one disability. A recent review originating from Japan alerts of the lack of protocols to improve long-term treatment outcomes and outlines a requisite paradigm shift from a disease-oriented approach to a problem-solving management [56]. This is also called better cancer support for patients and their families.

## 6. The Financial Toxicity of Oral Mucositis

A patient facing a cancer diagnosis will undergo numerous and important medical costs that will have an effect that has recently been referred to as financial toxicity [57]. Household adaptation to the economic burden of cancer does not necessarily have a positive result for the cancer patient and his/her family. Furthermore, oral mucositis has been reported to be one of the most frequent and expensive secondary events induced by cancer treatment.

The costs derived from oral mucositis in hematopoietic stem cell transplantation can range from USD 112,447 to USD 29,921,414 per patient [58]. The hospitalization and extended monitorization of patients with oral mucositis also put pressure on the already high economic burden of cancer treatment for institutions and governments. 

Finding safe measures for oral mucositis looks like a necessity not only for cancer patients but also for the rest of the actors involved, including health systems.

## 7. The Value of Patient-Reported Outcome Measures (PROMs) in Mucositis

When it comes to oral mucositis, divergences between generally lower-severity pictures perceived by oncologists and what patients experience have been reported [59]. These discordant perspectives have been noted in other fields of medicine wherein the mucosa is the main actor, such as gynecology and, in particular, in conditions such as breast cancer and the genitourinary mucosal secondary effects of breast cancer therapy, including adjuvant therapy that leads to cancer-therapy-related menopause [60,61,62]. PROMs are especially useful in the field of oncology wherein physicians and healthcare professionals tend to underestimate symptoms that affect patients’ quality of life. Medical teams focus on survival and the risk of the recurrence of the cancer disease. This diverts attention from collateral symptoms that nevertheless severely affect a patient’s quality of life or even lead to the abandonment of the treatment [63]. Therefore, questionnaires based on patients’ reports, also called PROMs, have become increasingly important.

It is important that PROMs questionnaires implemented for the assessment of oncology patients include signs of the existence or not of the cohort of emotional and physical symptoms that are highly prevalent, namely, sadness, anxiety, emotional distress, or social withdrawal. It is also advisable that the patients be able to report signs and symptoms such as the existence and degree of dysphagia, xerostomia, trismus, dysgeusia, appetite changes, sleep quality, as well as difficulty in speaking and communicating [64,65,66]. However, poor compliance to long questionnaires should be anticipated, and it may be preferable to choose a PROMs with a smaller number of questions.

It has been reported that symptoms of cancer treatment toxicity subjectively perceived by a patient are at risk of being underestimated by the clinician in the range of 40–70%. This occurs even in data collection during randomized studies, which is why the incorporation of ideally validated PROMs is emphasized in clinical studies [67].

Approaching patient needs and preferences from a shared patient–physician decision-making approach is now greatly promoted [68] and has been linked to greater independence, effectiveness, and efficiency of treatment as well as increased adherence to treatment [69,70,71]. 

## 8. Measures to Improve Oral Mucosal Resilience in Cancer Treatment: New Perspectives and Future Directions

It has been reported that patients experiencing oral pain show a lower salivary flow compared with controls [72]. Oral moisturizers and saliva stimulants for relieving the long-term oral complications of cancer treatment are the first-line treatment and have had a presence in guidelines and experts’ recommendations. However, there is concern about products based on acidic formulations regarding their erosive potential in already susceptible patients. In this respect, a non-acidic composition has been included in numerous guidelines and systematic reviews for the proven benefits in dry mouth patients [73,74,75,76,77,78,79].

A clinical trial performed in H&N cancer radiated patients, with the primary outcome being a decrease in symptoms after the use of the previously described composition, demonstrated a significant improvement in long-term symptoms, physical, social, and personal, and pain, as well as in the quality-of-life parameters analyzed, limitation in eating, limitation in enjoying food, limitation in speaking, and limitation due to dryness. Patients reported fewer interruptions during sleep [80]. No side effects nor adverse events were reported. 

Although oral mucositis was not included as an outcome, patients experienced a statistically significant pain reduction even though only 40% of the patients showed increased salivary flow, while 60% did not. Therefore, another mechanism for pain amelioration must be implicit. 

Partial unpublished data from an ongoing interventional study registered at ClinicalTrials.gov (https://clinicaltrials.gov/ct2/show/NCT05635929 (26 May 2023)) in 50 H&N cancer patients with a mucositis severity outcome during the first 6 weeks of treatment and follow-up after 6 months have pain as an outcome and oral mucositis severity. The use of PROMs in conjunction with a mucosal topical non-acidic lipophilic composition enriched with a potent natural antioxidant, under the name saliactive^®^, seems to be in line with the accumulating evidence of reactive oxygen species (ROS) pathways and their mediation of the inflammatory cascade that may be behind a good part of the complex pathobiology of chemo/radiotherapy-induced oral mucositis [81]. Additional health benefits of naturally occurring antioxidants have been extensively documented [82]. In the acute phase, when measuring oral mucositis (OM), the percentage of patients that develop OM is 89.7% in line with previously reported data. However, the percentage of severe OM (grades 3–4) patients is 34.4% compared with the 66% severe OM patients reported by Elting et al. [83], the 65% by Elad et al. [84], and the 29% by Vera-Llonch (Vera-Llonch M et al. Oral mucositis in patients undergoing radiation treatment for head & neck carcinoma. Cancer 2006). Only 3.4% of patients have grade-4 OM, while treatment interruptions have happened in only 13.3% (Table 2). In the acute phase, which has already been completed in the aforementioned interventional study, a convergence was detected between PROMs reported by patients (subjective information) and mucositis grades observed by the clinicians (objective information), independent of gender and age, reducing the gap oftentimes found between patients’ and clinicians’ reports [85]. The data show significant pain reduction after 1 month of use of a mucosal topical gel, along with significant improvements in recreation, appearance, saliva, and mood analyzed 6 months after treatment.

In a previous study, the associations between severe OM and adverse clinical outcomes such as hospitalization and treatment interruptions were established. Furthermore, the authors concluded that the higher the grade of OM, the longer the interruptions [86].

The role of the oral microbiome in the onset of oral dysbiosis because of cancer therapy and its relation to the cancer therapy response has been described before. The potential benefit of improving oral dysbiosis in this group of patients needs to be undertaken with support measures as dental interventions are restricted during the duration of cancer treatment. In this respect, a fresh-from-the-press controlled, double-blind, and multicenter randomized clinical trial, declared at ClinicalTrials.gov (https://clinicaltrials.gov/ct2/show/NCT05463484 (accessed on 19 July 2022)) as part of the Stop Dysbiosis Project, has shown statistically significant superior clinical outcomes in oral clinical parameters in patients with oral dysbiosis with the use of a microbiome-boosting toothpaste containing extra virgin olive oil (EVOO), xylitol, and betaine. While the control and placebo groups resulted in significant decreases in pH levels, the tested group kept pH levels at the physiologic value, contributing to oral eubiosis [87]. A low pH has been documented at dysbiotic sites with inflammation or cell destruction [88]. Furthermore, an acidic pH has been associated with inflammasomes and an abundance of proinflammatory cytokines, while on the contrary, physiological pH maintenance has been reported to inhibit the activation of proinflammatory pathways. A new body of thinking considers pH modulation a novel anti-inflammatory approach [89].

The results found in this RCT, as hypothesized by the authors, can be attributed to multiple mechanisms, namely, the action of numerous phenolic compounds with strong anti-inflammatory and antioxidant capacities [90,91,92,93,94], a natural osmo-protecting amino acid that is able to interfere with a wide range of inflammation-related circuits [95,96], and prebiotic proinflammatory-cytokine inhibition [97]. Early detection and reversion of subclinical microbiome changes in human mucosal tissues may be of help if, as it seems, an imbalanced microbiome can influence the severity and course of OM [37]. Oral microbiota dysbiosis, in a recent review, was said to accelerate the development and onset of oral mucositis through factors influencing oral mucosal microbiota shifts such as smoking, radiotherapy, stem cell transplants, inflammatory factors released into the oral cavity, genetic factors and epigenetic factors, oral mucosal barrier breakage, and the combination of all or some of the above-mentioned [98].

Dysbiosis can be easily understood as a result of cancer’s game-changing condition. However, a question has been posed regarding women with ovarian cancer and dysbiosis in the vaginal mucosa. An imbalanced vaginal microbiota has been linked to an increased risk of ovarian cancer [99]. However, does vaginal dysbiosis occur before or after ovarian cancer? The unique lactobacillus-predominant signature in young and healthy women is an opportunity to conduct research to clarify the readiness of a disrupted microbiome to return to homeostasis, as a prerequisite in the vagina is the existence of low diversity and low pH (4–4.8). Interestingly, it has been hypothesized that a vagina poor in lactobacillus species or otherwise with vagina dysbiosis that develops inflammation and mucosal barrier breakage can facilitate papillomavirus penetration and tissue damage patterns associated with viral infection and, eventually, cervical cancer [100]. The finding that younger women with BRCA mutations were almost three times less likely to have healthy vaginal microbiota than those without the mutation can only be explained if the changes in the microbiome are in some way dictated by the genetics of an individual. Moreover, women with relatives diagnosed with ovarian cancer were themselves more prone to vaginal dysbiosis. 

An ongoing randomized blind comparative experimental clinical trial in patients with a history of malignant breast cancer declared at ClinicalTrials.gov (https://clinicaltrials.gov/ct2/show/NCT05585476 (accessed on 1 December 2022)) will help to elucidate the potential of a synergistic and symbiotic interaction with the host at the genital mucosa level once it has lost estrogen protection. All attempts to minimize cancer-therapy-related effects on the genitourinary mucosa in breast cancer survivors that should be promoted as therapeutic strategies are limited in this group of patients, as the applications of estrogens and/or selective estrogen receptor modulators are either non-indicated or highly restricted [101,102,103].

Moreover, the complexity behind OM management considered as unexpected toxicity from usual oral hygiene ingredients or common oral antiseptics should not be disregarded in cancer patients. In this sense, The Mucositis Oral Guidelines Leadership Group (MASCC/ISOO; the Mucositis Guidelines Group of the Multinational Association of Supportive Care in Cancer and the International Society of Oral Oncology) updated its guidelines in 2021 by means of a systematic review through which they were able to identify the interventions with the highest evidence of being the most effective [104].

Surprisingly, chewing gum is not recommended for oral mucositis in the above mucositis guidelines. 

Several expert opinions complement these guidelines with the following recommendations: dental evaluation and professional treatment prior to cancer therapy with the desire to reduce the risks of local or systemic infections of odontogenic origin, and patient education on the benefits of basic oral care to improve self-care and adherence to oral care protocols. Special attention should be implemented prior to and during treatment, including rinsing with saline and sodium bicarbonate, which, despite being inert, improve overall cleaning, help maintain oral hygiene, and improve patients’ well-being. 

The implementation of oral care protocols is beneficial for the prevention of oral mucositis during QT, head and neck RT, and hematopoietic stem cell transplantation (HSCT). It is worth mentioning that it is suggested not to use chlorhexidine in patients treated with radiotherapy in the head and neck area. Furthermore, in a case-control study on risk factors regarding the onset of osteoradionecrosis of the jaw in radiated head and neck patients, the use of chlorhexidine multiplied the risk 1.28-fold and root scaling performed within the previous 2 weeks 2.43-fold [105].

Careful timing of interventions is advisable as well as the preferential use of natural ingredients such as honey, saliactive^®^, and glutamine as part of the emerging evidence of the potential of natural topical approaches for counteracting the toxicity in human mucosa [106,107,108,109]. Nevertheless, an important consideration for honey in the management of oral mucositis is its diabetogenic side effect.

Attention should be paid to the pro-metastatic evidence that some lipids, different from the oleic or linoleic acid in extra virgin olive oil, such as palm or palmitic oil have shown. This is of special relevance in patients suffering from oral cancers, as the authors showed that short dietary exposure to palmitic acid from palm oil in mice with oral carcinomas led to long-term stimulation of metastasis. A description of the pro-metastatic pathway of palm oil, when part of a diet even in small amounts, is a source of worry due to the potentially negative complex interactions between palm oil low-quality lipids and H&N cancer patients [110].

Efforts are being made to identify valid biomarkers such as pro-inflammatory cytokines for the early detection of patients that are especially susceptible to oral mucositis. The complexity of microorganism metabolomics, as well as the fluctuating behavior of cytokines, pro and against inflammation, hinders the prospect of a pure microbiome dysbiosis pattern being claimed as the initiator of oral mucositis, even though a more resilient microbiome with a lower inflammatory potential seems to protect from developing oral mucositis [111].

## 9. Conclusions

Oral mucositis is a complication that appears during chemo and/or radiotherapy treatment and involves the breakdown of the mucosal barrier, risk of systemic infection, and substantial pain, which can lead to the premature termination or remodeling of treatment and the interruption of treatment and eventually may affect a patient’s survival. Chronic side effects related to cancer and cancer therapy and chronic pain associated with oral mucosa also affect the quality of life of cancer survivors and challenge their health caretakers.

The increasing prevalence of cancer with more patients with access to cancer therapies, and more cancer survivors for more years, make it necessary to minimize the side effects of cancer treatment toxicity on the oral mucosa.

New approaches with synergistic efforts between basic science, clinicians from different disciplines, and translational research are needed to limit the toxicity of cancer treatments to reduce the incidence and severity of oral mucositis and provide better cancer support. 

New topical antioxidant microbiome-boosting strategies to minimize cancer therapy adverse effects in the oral mucosa seem to offer a window of opportunity for patients during oncological treatment in the context of oral mucositis.

## Figures and Tables

**Table 1:** Mucositis incidence depending on cancer type and mode of therapy. Data extracted from Pulito et al. [12].

	Mucositis Incidence
Chemotherapy-treated patients	30–40%
Hematopoietic stem cell transplantation (HSCT)	60–85%
H&N radio + chemotherapy	90%

**Table 2:** Incidence of severe oral mucositis (grades 3–4) in H&N.

Source	Severe Oral Mucositis (Grades 3–4)
Elting et al. [83]	66%
Elad et al. [84]	65%
Vera-Llonch et al. [86]	29%

## Data Availability

https://clinicaltrials.gov/ct2/show/NCT05635929 (accessed on 19 July 2022); https://clinicaltrials.gov/ct2/show/NCT05463484 (accessed on 19 July 2022); https://clinicaltrials.gov/ct2/show/NCT05585476 (accessed on 19 July 2022).
